# Effects of *Larrea nitida* nanodispersions on the growth inhibition of phytopathogens

**DOI:** 10.1186/s13568-023-01605-z

**Published:** 2023-09-21

**Authors:** Felipe Rocha, Rodrigo José Nunes Calumby, Laura Svetaz, Maximiliano Sortino, Márcia Cristina Teixeira Ribeiro Vidigal, Valeria Alina Campos-Bermudez, Sebastián Pablo Rius

**Affiliations:** 1 Centro de Estudios Fotosintéticos y Bioquímicos (CEFOBI-CONICET), Universidad Nacional de Rosario, Suipacha 531, S2002LRK Rosario, Argentina; 2 Facultad de Ciencias Bioquímicas y Farmacéuticas, Farmacognosia, Universidad Nacional de Rosario, Suipacha 531, CP 2000 Rosario, Argentina; 3 Food Science Department, Federal University of Vicosa, Peter Henry Rolfs Av., Viçosa, MG 36570-900 Brazil

**Keywords:** Natural antimicrobial extract, Solid dispersion, Biotechnology, Phytopathogens

## Abstract

*Larrea nitida* Cav. (*Zygophyllaceae*) is a plant endemic to Argentina and Chile, and its extract has been studied over the last years due to the presence of antimicrobial agents that can be used to control the growth of some pathogens in agriculture. However, the extract is highly hydrophobic, which strongly affects its fungicidal activity in aqueous media. In this sense, the solid dispersion technique was used to produce *L. nitida* extract nanodispersions with polyethylene glycol (PLE) and with polyethylene glycol and zinc acetate (PZLE). In order to further evaluate the activity of the extract in PLE and PZLE, blank nanodispersions containing only polyethylene glycol (PEG) and zinc acetate (PZ) without the addition of the extract were also produced. The fungicidal activity of the water-soluble nanoparticles was evaluated at different concentrations (0.037–0.110 g.mL^−1^). In general, the nanoparticles were successfully produced on a nanometric size and presented a significant inhibitory activity on the growth of the pathogens *Fusarium oxysporum* and *Fusarium verticillioides* in aqueous media. Compared to PLE, PZLE presented increased fungistatic activity, possibly due to their increased solubility in water. Even though their application in agriculture should be further investigated, the nanodispersions present great potential to be applied as a green biotechnological tool.

**Supplementary Information:**

The online version contains supplementary material available at 10.1186/s13568-023-01605-z.

## Introduction

Pathogens in crops can cause significant losses in agriculture, consequently, many chemicals are currently used to prevent the growth of pathogens and fungi in crops (Al-Ani and Furtado 2020). However, the use of these compounds has been a concern due to the fact that agrochemicals are very toxic to the human body and can heavily pollute the environment (Gupta and Crissman 2013). Moreover, agrochemicals can heavily alter the soil microbiota, which affects microorganisms that play important roles in the growth of plants (Khan et al. 2020). In this sense, alternative sources to conventional agrochemicals are increasingly demanding.

*Larrea nitida* Cav. (*Zygophyllaceae*) is a plant endemic to Argentina and Chile, known for its antioxidant and fungicidal activity (Moreno et al. 2020a). Studies have shown that the extract from *L. nitida* can be applied for food packaging as a foodborne antimicrobial agent (Moreno et al. 2020a) and also for the inhibition of the growth of human pathogens (Butassi et al. 2015, 2019). Essential oils and/or natural extracts have been often used to produce materials with antimicrobial activity (Saraiva et al. 2021; Barbălată-Mândru et al. 2022; Marques et al. 2022). *L. nitida* extract is rich in antimicrobial compounds and organic acids, such as epoxylignans, ferulic and nordihydroguaiaretic acids, and phenolic compounds. However, data regarding a further detailed composition of the extract is scarce in the literature. It is known that the hydrophobic nature of the extract can make it challenging to be applied in agriculture without the use of organic solvents (Agüero et al. 2011). The use of organic solvents can contribute to releasing compounds that are toxic to the human body, and can also increase the risk of pollution of the environment and water sources. Furthermore, some organic solvents can alter the soil microbiota by acting as antimicrobial agents per se (Dick 2006; Dyrda et al. 2019). It is believed that a novel technology, known as solid dispersion, can be used to produce a water-soluble bionanomaterial containing *L. nitida* extract with biodegradable polymers as a biotechnological tool for the control of pathogens in agriculture.

The solid dispersion method consists of using a polymer as a solid solvent for the compound or extract of interest. This technique has been widely used with promising results regarding the improvement in water solubility of hydrophobic bioactive compounds (Vasconcelos et al. 2007; Rocha et al. 2018; Manzoor et al. 2022). Silva et al. (2017) used the solid dispersion technique to encapsulate lutein and reported an increased solubility of 43 times for the nanoparticles in water when compared to lutein in its pristine form. In our study, polyethylene glycol (PEG) and zinc acetate were chosen as wall materials to produce nanodispersions of *L. nitida* extract. PEG is a non-toxic and biodegradable polymer, which contains both hydrophobic and hydrophilic groups in its chemical structure (Huang et al. 2019; Shi et al. 2021). Several studies have reported promising results with the use of high molecular weight polymers as wall materials to produce nanodispersions (Lin et al. 2016; Reboredo et al. 2021; Rocha et al. 2023). Data available in the literature show that zinc acetate can be used to produce nanoparticles on a nanometric scale for agricultural purposes due to its fungicidal activity (Sun et al. 2018; Saravanakumar et al. 2020; Faisal et al. 2021). Indeed, the complexation of materials with fungicidal activity seems to be an interesting approach.

There are several species of fungi of interest in agriculture. For example, *Trichoderma harzianum* is a soil-borne filamentous fungus known for their ability to produce metabolites that play an important role in the growth promotion of plants, such as tomatoes, maize, cucumber, and others (Stewart and Hill 2014; Joo and Hussein 2022; Natsiopoulos et al. 2022). Furthermore, some species produce metabolites that possess antimicrobial activity and can be used to inhibit some pathogens in plants (Nieto-Jacobo et al. 2017; Xie et al. 2021). On the other hand, *Fusarium oxysporum* is a pathogen responsible for great losses in agriculture. This fungus attacks the vascular system of plants, causing vascular wilts in several crops, such as tomatoes, potatoes, bananas, and others (Kant et al. 2019). Species *Fusarium verticillioides* produces toxins that infect grains, making them unsafe for human consumption due to their toxicity in the human body, and is also responsible for kernel and ear rot in maize (Bacon et al. 2008; Beccaccioli et al. 2021).

In this sense, the aim of this study is to investigate, in aqueous media, the in vitro fungicidal activity of *L. nitida* nanodispersions produced with PEG and with a mixture of PEG and zinc acetate as wall materials, in contrast with the use of agrochemicals for this purpose. The inhibitory effects of the nanodispersions on the growth of *T. harzianum 10BR1*, *F. oxysporum*, and *F. verticillioides* were evaluated. It is expected an increased solubility of the nanodispersions in aqueous media and the maintenance of their fungicidal activity.

## Materials and methods

### Materials

Methanol (99.8%, Cicarelli, Argentina) was used to obtain *L. nitida* Cav. (*Zygophyllaceae*) extract. PEG (6000 g.mol^−1^, Anedra, Argentina), zinc acetate (Cicarelli, Argentina), and ethanol (99%, Cicarelli, Argentina) were used to produce the nanodispersions. Dimethyl sulfoxide (DMSO, Merck, Germany) was used to dilute the extract for the ultraviolet-visible spectroscopy analyses. Potato Dextrose Agar (PDA) was used as the culture medium for the microorganisms. The strains of the fungi *F. oxysporum* strain CCC 186-06, *F. verticillioides* strain P364, and *T. harzianum* 10BR1 strain CCC 115 − 23 were obtained from the sample collection of the Center of Photosynthetic Studies and Biochemists (CEFOBI) at the Universidad Nacional de Rosario. All the solvents used for the following experiments were analytical grade.

### Production of ***L. nitida ***extract

Aerial parts of *L. nitida* Cav. (*Zygoph*yllaceae) were collected in Bauchaceta city, San Juan, Argentina. The plant was previously identified by Dr. Gloria Barboza, from the National University of Córdoba, Argentina, and a voucher specimen was deposited in the herbarium of the Botanic Museum of Córdoba (CORD 1335).

The extract was obtained according to the methodology proposed by Moreno et al. (2020a), with some modifications. Briefly, 100 g of the aerial parts of the plant were ground and diluted in methanol under magnetic stirring for 24 h. After filtration with Whatman filter paper number 1, the solvent was removed in a rotary evaporator, resulting in an extraction yield of 50.8% (w/w). The extract was stored at −20 ºC in absence of light until further analysis.

The characterization of *L. nitida* extract has been published elsewhere. It is known that the extract contains some fungicidal compounds, such as ferulic and nordihydroguaiaretic acids, epoxylignans, and phenolic compounds (Agüero et al. 2011). The extract was lyophilized for the following experiments.

### Production of nanodispersions

The production of nanodispersions by the solid dispersion technique was carried out with a proportion of 1:10 (w/w) of extract and wall materials, respectively. This formulation has been reported in the literature with promising results regarding an improvement in water solubility of hydrophobic bioactive compounds (Silva et al. 2017; Rocha et al. 2018; Sá et al. 2019). In this sense, *L. nitida* extract (LE) nanodispersions were prepared according to the methodology proposed by Rocha et al. (2022), with some modifications. To further assess the effects of the extract, blank nanodispersions (PZ) were produced only with zinc acetate (90 mg) and PEG 6000 g.mol^−1^ (90 mg). PLE nanodispersions were produced with LE (18 mg) and 180 mg of PEG (6000 g.mol^-1^). For PZLE, 90 mg of zinc acetate was homogenized with 90 mg of PEG (6000 g.mol^-1^), and 18 mg of LE. After dissolution for 1 h under magnetic stirring at room temperature in ethanol, all the solutions were placed in an ice bath and sonicated in an ultra-turrax (120 W) for 3 min under pulses conditions of 30 s on and 10 s off. The nanodispersions were then dried in an oven at 60 ºC for 24 h. For the following experiments, all the solutions containing the nanodispersions were centrifuged at 5000 g for 10 min, and the supernatant was stored at 4 ºC in absence of light for further analyses.

### Particle size and ζ-potential measurements

The particles’ hydrodynamic diameters and the ζ-potential were determined with Zetasizer Nano-ZS (Malvern Instruments, Worcestershire, UK) in deionized water at a pH of ~ 6.7, the typical soil pH for crops. The size distribution of nanodispersion was characterized by using Dynamic Light Scattering (Zetasizer Nano-ZS, Malvern Instruments). For determination of the average size of the particles, the analysis was carried out at 173º scattering using a 632.8 nm wavelength excitation. The samples were diluted in a proportion of 1:10 in deionized water and ultrasonicated for 10 min prior to analysis to avoid multiple scattering effects and the experiments were performed at room temperature.

### Ultraviolet-visible spectroscopy

The ultraviolet-visible spectra of the samples were obtained with an ultraviolet-visible spectrophotometer (NanoDrop, Thermo Fisher Scientific, USA). LE was first diluted in DMSO and then completed with water yielding a final concentration of 10% of DMSO (v/v). The extract and the nanodispersions were diluted in water to yield a final concentration of 0.025 g.mL^−1^ and 0.275 mg.mL^−1^, respectively. The concentration of extract in water used for this experiment corresponds to the concentration of extract (9.1% w/w) present in the nanodispersions, as stated in the methodology described for the production of the nanoparticles.

### Attenuated total reflectance - fourier transform infrared (ATR-FTIR)

The chemical interactions between the constituents in the nanodispersions were evaluated by an ATR-FTIR (GladiATR, Pike Technologies, USA) with a resolution of 4 cm^−1^ from 900 to 4000 cm^−1^ by coadding 20 scans. The infrared spectra of the physical mixtures (PM) were evaluated by manually mixing the constituents of the nanodispersions in the same mass proportion used in the methodology for the production of the nanoparticles.

### In vitro fungicidal activity

The fungicidal activity of the nanodispersions was determined according to the agar diffusion methodology, proposed by Poggio et al. (2017), with some modifications. PDA was used as the culture medium, and the solutions containing the nanodispersions in water were mixed with a dispersion containing 10^4^ spores of fungi in proportions of 1:1, 1:2, and 1:3, respectively, resulting in final concentrations of 0.110, 0.055, and 0.037 g.mL^−1^. The same dilution proportions were applied for the control treatments with water for each experiment.

The fungicidal activity of the extract in aqueous media (EXT) was also evaluated. Since the concentration of fungicides plays a role in the fungicidal activity, the concentration of extract in water used for the experiment (0.010 g.mL^−1^), which is equivalent to the amount of extract present in the nanodispersions at the maximum concentration evaluated in this study (0.110 g.mL^−1^). Each plate contained each treatment (water, PZ, PLE, and PZLE) and (water, EXT, PZ, and PZLE) placed in 4 equidistant points in a plate.

For *T. harzianum* 10BR1, the diameter of the colonies treated with water, EXT, PZ, PLE, and PZLE was measured for 72 h every 24 h. For the colonies with *F. oxysporum* and *F. verticillioides*, the diameters were measured after 48 h for 96 h. The experiments were done in triplicate and the results were expressed in growth rate (%) relative to the maximum diameter obtained for the control with water only.

### Statistical analysis

All the experiments were performed in triplicate. ANOVA one-way using *p* < 0.05 was carried out with R statistical software (v. 4.2.0; R Core Team 2022). When appropriate, the significant differences between the means of the treatments with water, EXT, PZ, PLE, and PZLE were determined by Tukey’s test.

## Results

### Particle size and ζ-potential measurements

**Fig. 1:**
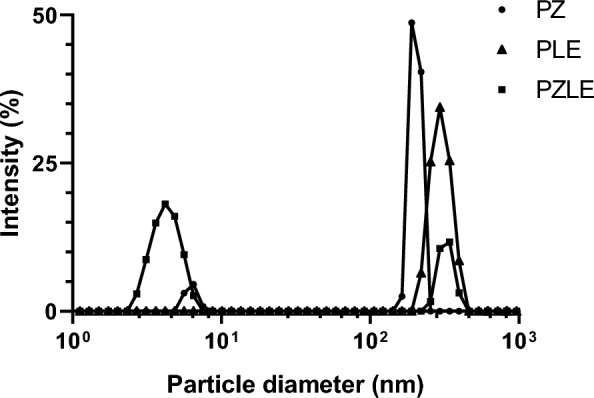
Intensity (%) as a function of particle diameter obtained by dynamic light scattering measurement of each formulation

In the analysis of particle size (Fig. 1), it was possible to observe that PZ presented two intensity peaks. 8% of the total amount of particles present an average size of 6.3 ± 0.6 nm and can be attributed to the fraction of zinc acetate dispersed in ethanol that was transformed into nanoparticles. The particles with an average size of 203 ± 16 nm can be related to the presence of dispersed PEG, corresponding to 92% of the total. Importantly, PZLE presented the same behavior regarding the size distribution, but with a different particle size proportion, where 73% of the particles present an average nanometric size of 4.3 ± 0.9 nm, and the other 27% size of 324 ± 36 nm.

Regarding the ζ-potential, PZ presented values of 31.3 ± 3.1 mV, PLE of −23.2 ± 3.7 mV, and PZLE of −6.2 ± 2.1 mV. Rocha et al. (2022) reported the same behavior regarding the ζ-potential for betalains nanodispersions produced with PEG only and with PEG and chitosan. According to the authors, the addition of chitosan significantly affected the ζ-potential of the particles.

### Ultraviolet-visible spectroscopy

**Fig. 2:**
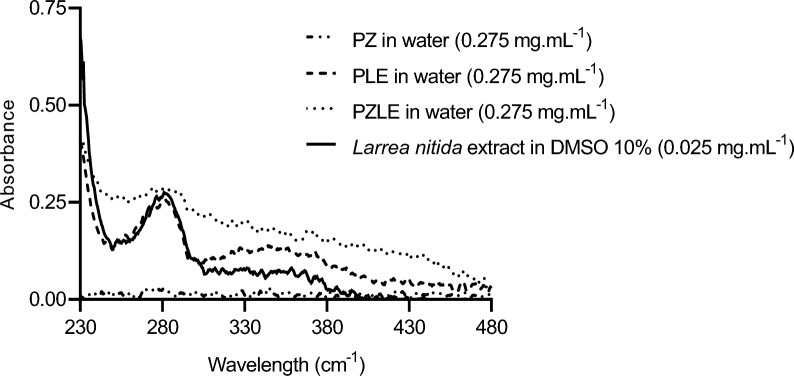
Ultraviolet-visible spectra obtained for PZ, PLE, and PZLE and for the extract diluted in DMSO 10%

LE contained many compounds that overlap the absorbance intensity measured in some specific wavelengths, especially organic acids and phenolic compounds. In general, these compounds present absorbance peaks at around 230 and 300 nm (Fig. 2) (Horbury et al. 2016; Kaeswurm et al. 2021). The spectrum obtained for the extract in DMSO 10% was very similar to the ones obtained for both PLE and PZLE in water. The absorbance obtained in a broad range of the spectrum for the nanodispersions can be explained by the presence of nanometric particles, meaning that these particles are more dispersed in water, thus, presenting more concentration of absorbing species. It is known that particle size strongly plays a role in the solubility of the materials, in the sense that smaller particles present an increased surface area of contact with the solvent, which strongly improves their solubility (Sun et al. 2012). Zinc oxide presents an absorbance peak at ~ 310 nm, which is not visible in the spectrum obtained for the blank nanoparticles (PZ), probably due to its low concentration (Fig. 2) (Zaki et al. 2021).

**Fig. 3:**
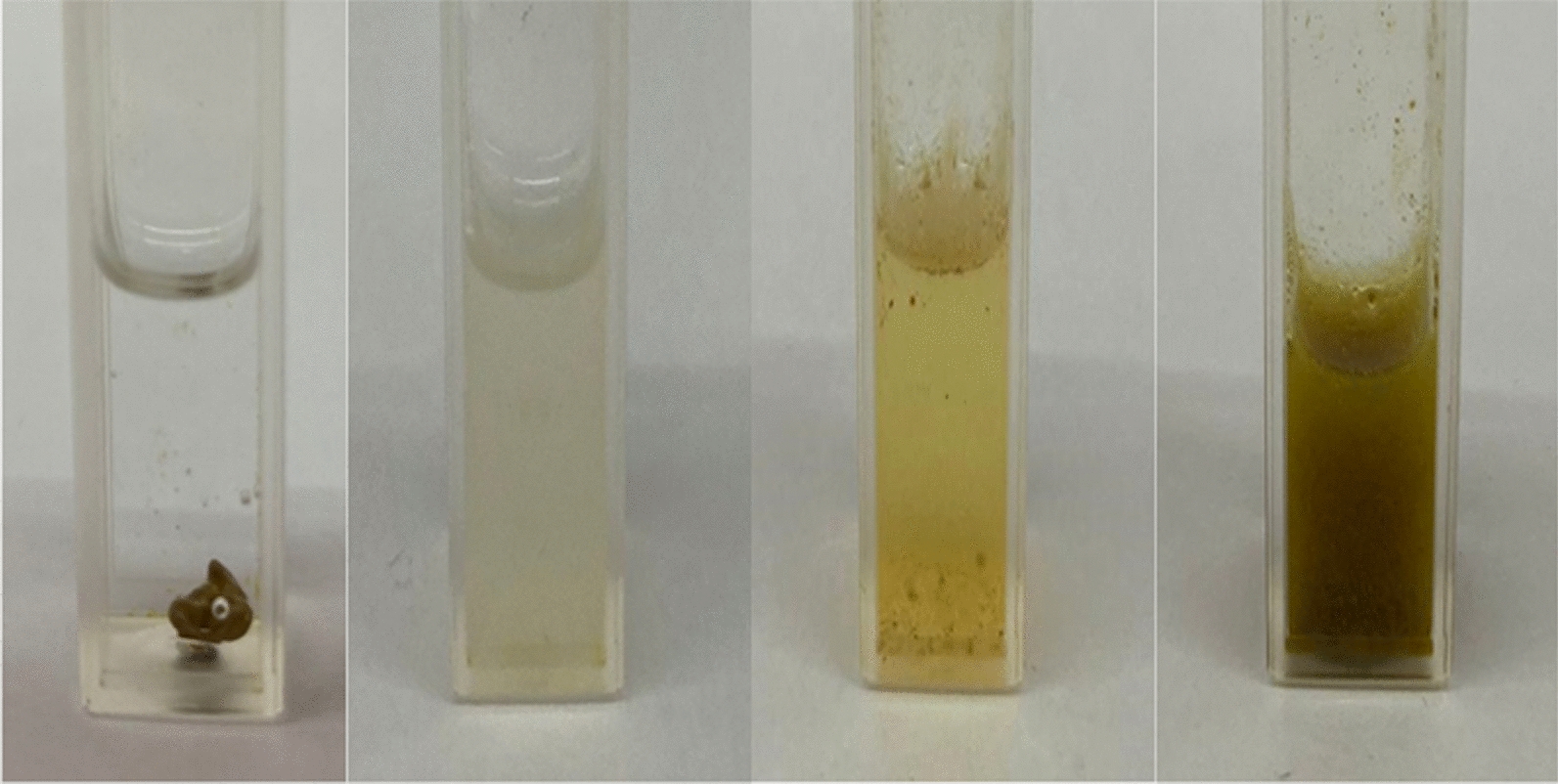
LE (0.010 g.mL^−1^) and PZ, PLE, and PZLE (0.110 g.mL^−1^) in water, from left to right, respectively

It is not possible to see the spectrum of LE in water (data not shown) due to its low solubility in water (Fig. 3). Therefore, the results show that the solid dispersion technique significantly increased the solubility of the extract in aqueous media (Fig. 3).

### Attenuated total reflectance - fourier transform Infrared Spectroscopy (ATR-FTIR)

**Fig. 4:**
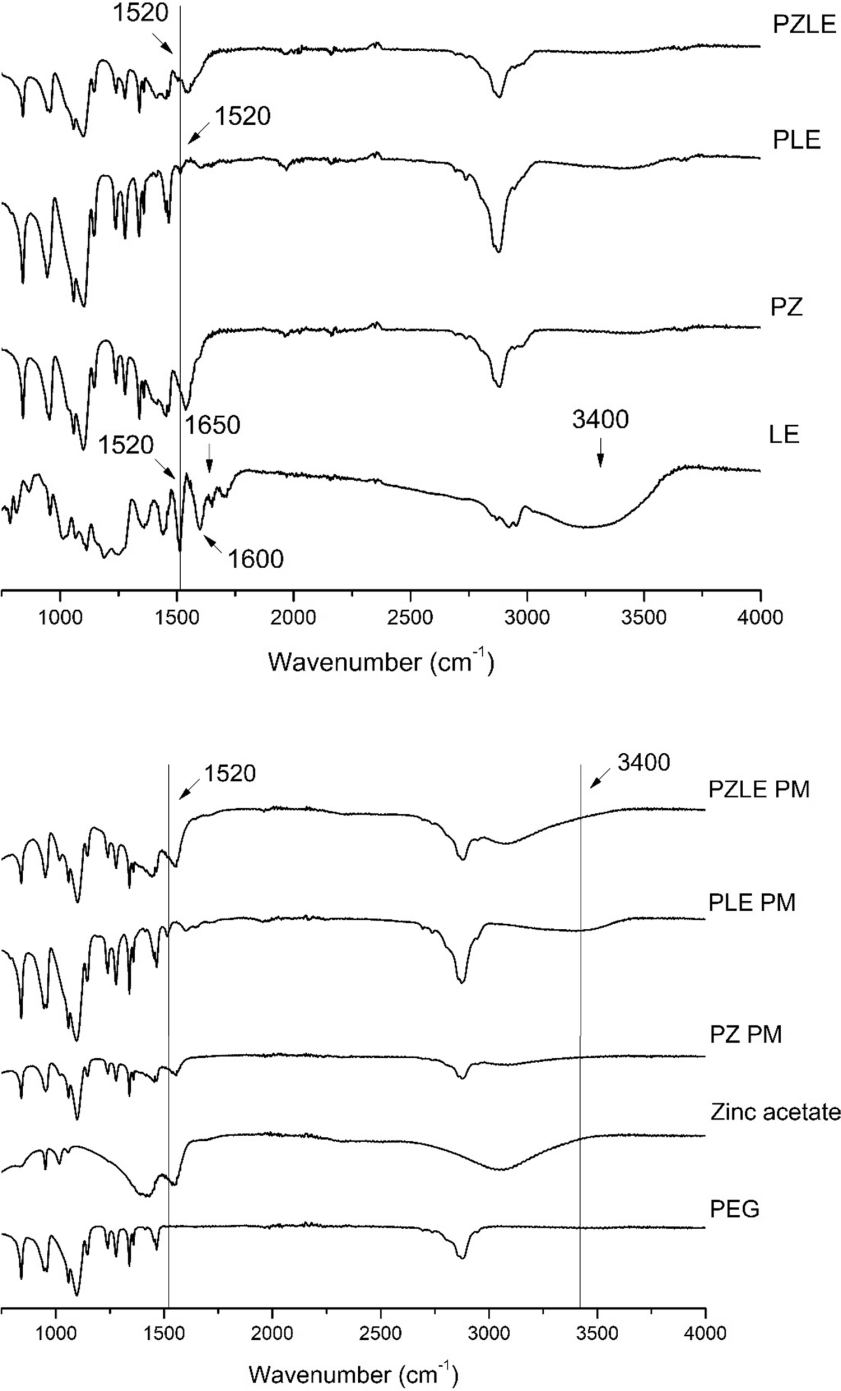
FTIR spectra obtained for PEG, zinc acetate, PZ, PLE, and PZLE nanodispersions (top) and for the physical mixtures (PM) of the constituents of PZ, PLE, and PZLE (bottom)

The ATR-FTIR spectrum obtained for LE presented a broad absorption band at 3400 cm^−1^, which can be attributed to O-H stretching groups (Tutunchi et al. 2019). The peak at ~ 1520 cm^−1^ is related to lignin skeletal bands in aromatics, whereas the bands at ~ 1600 and 1650 cm^−1^ represent the C = C stretching in aromatics and alkenes, respectively (Fig. 4). The results corroborate the characterization of the extract published elsewhere, which indicates the presence of compounds that present these groups in their molecular structure, such as epoxylignans, ferulic and nordihydroguaiaretic acids (Agüero et al. 2011).

It was possible to observe that the spectra obtained for the physical mixtures were not significantly different from the ones obtained for the nanodispersions, despite the presence of the broad bands at ~ 3400 cm^−1^ in the spectra obtained for the physical mixtures, possibly due to the hygroscopic nature of the extract in its pristine form. Importantly, both PLE and PZLE nanodispersions presented a transmittance peak at ~ 1520 cm^−1^, which was not present in the spectra obtained for both PEG alone and PZ physical mixture, suggesting that it can be attributed to the presence of the extract in the nanodispersions.

FTIR results indicated that the chemical structure of the constituents of the nanodispersions was not significantly affected during their transformation into nanoparticles (Zhang et al. 2012). The attenuation of some characteristic bands of the extract in the spectra obtained for PLE and PZLE and their corresponding physical mixtures can be explained by the difference in the mass proportions of the constituents used to produce the nanodispersions.

### In vitro fungicidal activity

Tables 1 and 2, and 3 present the results regarding the fungicidal activity of the nanodispersions against the fungi *T. harzianum* 10BR1 (Table 1) and the pathogens *F. oxysporum* (Table 2) and *F. verticillioides* (Table 3).

**Table 1:** Inhibitory effects (%) on the growth of *T. harzianum* 10BR1 relative to the control with water

Growth inhibition (%) of *T. harzianum* 10BR1* after 24 and 48 h						
Sample	0.037 g.mL^−1^		0.055 g.mL^−1^		0.110 g.mL^−1^	
	Time (h)					
	24	48	24	48	24	48
PZ	2ª	0ª	3ª	0ª	3ª	0ª
PLE	0^b^	0ª	2ª	0ª	3ª	0ª
PZLE	3^a^	0ª	4^b^	0ª	7^c^	0ª

The experiments were performed in triplicate

^*^All the standards deviation were smaller than 5%

^**^Different letters mean that the treatments are significantly different

**Table 2:** Inhibitory effects (%) on the growth of *F. oxysporum* relative to the control with water

Growth inhibition (%) of F. oxysporum* after 48, 72 and 96 h									
Sample	0.037 g.mL^−1^			0.055 g.mL^−1^			0.110 g.mL^−1^		
	Time (h)								
	48	72	96	48	72	96	48	72	96
PZ	0ª	0ª	0ª	2ª	2ª	1ª	5ª	8ª	8ª
PLE	9^b^	8^b^	8^b^	10^b^	10^b^	11^b^	14^b^	14^b^	12^b^
PZLE	29^b^	28^c^	17^c^	38^c^	28^c^	20^c^	48^c^	36^c^	23^c^

The experiments were performed in triplicate

^*^All the standards deviation were smaller than 5%

^**^Different letters mean that the treatments are significantly different

**Table 3:** Inhibitory effects (%) on the growth of *F. verticillioides* relative to the control with water

Growth inhibition (%) of F. verticillioides* after 48, 72 and 96 h									
Sample	0.037 g.mL^−1^			0.055 g.mL^−1^			0.110 g.mL^−1^		
	Time (h)								
	48	72	96	48	72	96	48	72	96
PZ	0ª	0ª	0ª	3.5ª	1.5ª	1ª	5ª	7.5ª	8.5ª
PLE	8^b^	5^b^	4^b^	10^b^	12^b^	9^b^	16^b^	17^b^	13^b^
PZLE	37^c^	30^c^	19^c^	39^c^	31^c^	20^c^	43^c^	33^c^	24^c^

The experiments were performed in triplicate

^*^All the standards deviation were smaller than 5%

^**^Different letters mean that the treatments are significantly different

For all the concentrations evaluated, the nanodispersions presented a fungistatic activity. The fungistatic activity is related to the concentration necessary to inhibit the growth of a fungi colony, whereas the fungicidal activity is the sufficient dose to kill the microorganism (Hazen 1998).

In general, the nanodispersions’ fungistatic activity seems to be proportional to the concentration (Tables 1 and 2, and 3). PZLE seems to be more soluble than PLE, which corroborates the results obtained for their fungistatic activity in aqueous media. Moreover, the difference in the fungistatic activity between both formulations can also be due to the fungicidal activity of zinc compounds (Lipovsky et al. 2011). In fact, the fungistatic activity of PLE seems to remain predominantly constant even with an increasing concentration. Even though the inhibitory effects of PZ were almost neglectable regarding the growth of *F. oxysporum* and *F. verticillioides*, as the concentration of zinc increased, it was possible to notice a slight decrease in the diameter of the colony, especially at a concentration of 0.110 g.mL^−1^ (Tables 2 and 3). Therefore, the fungistatic activity of PZLE can be related to both the presence of zinc and especially LE in this formulation.

The inhibitory effects of PZLE at a concentration of 0.110 g. mL^−1^ was similar for both pathogens tested, with growth inhibition of 48% for *F. oxysporum* and 43% for *F. verticillioides* after 48 h of incubation. Importantly, after 96 h and even at small concentrations, PZLE presents strong fungistatic activity against the tested pathogens.

**Fig. 5:**
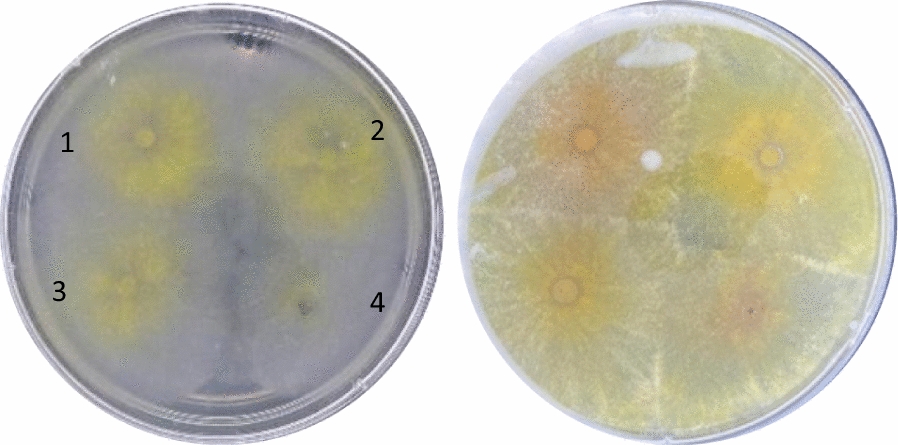
*T. harzianum 10BR1* after 24 and 48 h of incubation, from left to right, respectively, at a final concentration of 0.110 g.mL^−1^ of nanodispersions in water. **1** Treatment with water, **2** treatment with PZ, **3** treatment with PLE, and **4** treatment with PZLE

**Fig. 6:**
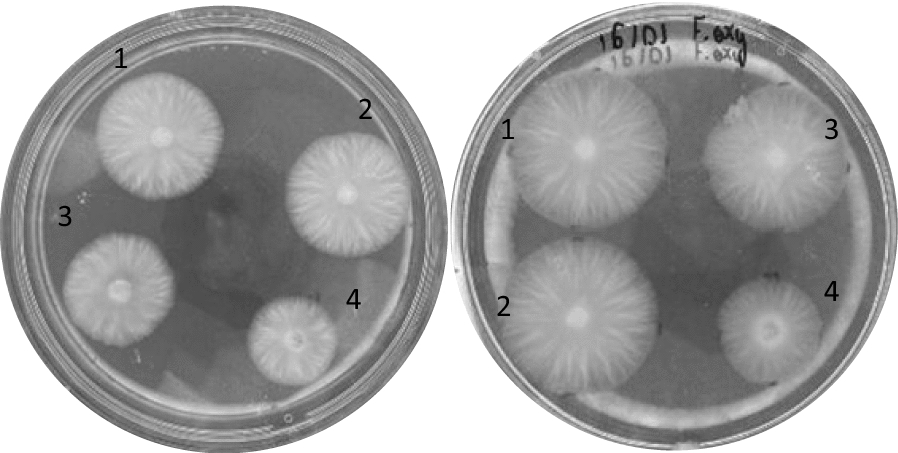
*F. oxysporum* after 72 h of incubation at a final concentration of 0.037 and 0.110 g.mL^−1^ of nanodispersions in water, from left to right, respectively. **1** Treatment with water, **2** treatment with PZ, **3** treatment with PLE, and **4** treatment with PZLE

**Fig. 7:**
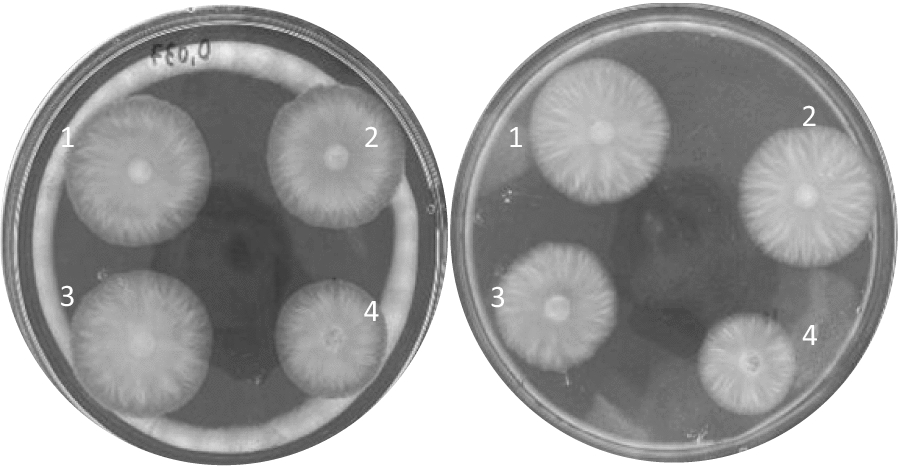
*F. verticillioides* after 72 h of incubation at a final concentration of 0.037 and 0.110 g.mL^−1^ of nanodispersions in water, from left to right, respectively. **1** Treatment with water, **2** treatment with PZ, **3** treatment with PLE, and **4** treatment with PZLE

It is known that some species of *T. harzianum* can be beneficial to some crops by assisting the growth promotion of plants (Vinale et al. 2009; Hang et al. 2022). Furthermore, according to Adams et al. (2007), *T. harzianum* can play in role in the phytostabilization of metal-contaminated sites. Even though there was a small inhibition in the first 24 h for all the concentrations evaluated, the results suggested that the fungus overcame the inhibition after 48 h at the maximum concentration evaluated in this study (Fig. 5). Since the growth inhibition of the fungi seems to be concentration-dependent, the results were not significant either for the concentrations of 0.037 and 0.055 g.mL^−1^ after 48 h of incubation, meaning that the nanodispersions did not significantly affect the growth of *T. harzianum* 10BR1. In fact, when comparing the growth inhibition of the pathogens *F. oxysporum* and *F. verticillioides*, the inhibitory effects of PLE and PZLE were significant even after 96 h of incubation (Figs. 6 and 7).

Pristine LE dispersed in water did not present any inhibitory effect regarding the growth of the fungi evaluated in this study when compared to the treatment with water only (Additional file 1: Figure S1).

The results indicate that water-soluble LE nanodispersions were successfully applied in vitro with great potential to inhibit the growth of pathogens of interest in agriculture, such as *F. oxysporum* and *F. verticillioides*. Importantly, the growth of *T. harzianum* was not significantly affected by the presence of the nanodispersions.

## Discussion

Several studies have reported the use of PEG and zinc acetate to produce nanoparticles containing bioactive compounds on a nanometric scale. Zinc acetate is widely used to produce zinc oxide (ZnO) nanoparticles, and the formation of these particles involves hydrolysis and condensation. The further dehydration of formed (Zn-OH)_2_ condensed with the compound of interest produces ZnO nanoparticles, releasing water as a result of the reaction (Mahamuni et al. 2019). In general, zinc oxide nanoparticles present an average size of 4–50 nm, significantly smaller compared to nanodispersions produced with PEG alone using the solid dispersion technique (Kanaze et al. 2006; Sun et al. 2018; Sayyar and Jafarizadeh-Malmiri 2019; Saravanakumar et al. 2020; Rocha et al. 2022).

In our study, the particles containing LE were successfully produced on a nanometric size. Since ZnO nanoparticles present a smaller average size, it is suggested that the particles within the range between 4 and 8 nm are mainly composed of the fraction of the extract complexed with ZnO. On the other hand, PLE presented larger particles with less polydispersity (310 ± 49 nm) compared to PZLE. The results corroborate the study carried out by Andishmand et al. (2017). The authors produced resveratrol nanocolloids with a mix containing PEG, pectin, and zinc chloride, and reported the same behavior regarding the size and the polydispersity of the particles, where a fraction of the particles present a significantly smaller average size compared to other particles present in the formulation.

It was possible to observe that the presence of LE significantly affected the ζ-potential of the nanodispersions, as PZLE presented values of −6.2 ± 2.1 mV compared to PZ, with a ζ-potential of 31.3 ± 3.1 mV. The ζ-potential is the measurement of the surface charge of the particles and can be affected by several conditions, such as pH, ionic strength, and the addition of electrostatically charged materials, such as salts. This property can affect the size of the particles due to electrostatic and/or repulsions between the particles. In fact, ζ-potential is a parameter related to the kinetic stability of particles in a system, however, many other factors can also affect the kinetic stability of colloidal systems, such as temperature, pH, viscosity, and others. In general, a ζ-potential higher than 30 mV in absolute value is considered a good reference (Awad et al. 2005; Xu et al. 2008). Even though the ζ-potential of PLE and PZLE is below 30 mV in absolute value, the steric repulsion can also play a role in the stability of colloidal systems. Steric repulsion is a result of the reduction in possible conformations between the molecules. In general, the presence of larger molecules in colloidal systems, such as PEG, can favor the occurrence of this phenomenon (Charles 1992).

When tested against phytopathogens, it was possible to observe that pristine LE did not present any fungistatic activity in water due to its limited solubility. As for the nanodispersions, the in vitro activity showed that the inhibitory activity of PZLE decreased over time, which can be explained by the mechanism of defense of the fungi in order to overcome the fungistatic activity. This mechanism of defense can include the release of metabolites that bind to specific target molecules, which can impair the viability of some antimicrobial compounds. Moreover, as the fungus grows, it acquires more resistance due to the formation of a more structured cell wall and increased production of defensive metabolites (Künzler 2018; Garcia-Rubio et al. 2020).

Water-soluble nanodispersions containing antimicrobial *L. nitida* extract were successfully produced, preserving their antimicrobial activity and allowing their application without the use of organic solvents. It was possible to observe that the fungistatic activity depends on the nanodispersions’ concentration, as expected. PZLE presented higher fungistatic activity than PLE, possibly due to its increased solubility in aqueous media and also to the presence of zinc compounds in the formulation. Importantly, the use of bionanomaterials to manage plant infection and/or disease has been intensively investigated over the last few years. In fact, nanomaterials can be absorbed more easily by membranes and cell walls due to their nanometric size, in addition to presenting an increased area of surface contact (Kulabhusan et al. 2022; Pattanayak et al. 2023). Even though the concentrations evaluated in this study did not provide a fungicidal effect, the inhibitory effects on the growth of the pathogens evaluated in this study allow more time to control the infestation of the microorganism in crops, which means more chances to recover the infected plant (Elmer et al. 2018).

This study provides new insights into the development of green biotechnological tools for agricultural purposes, providing new insights into the reduction in the use of agrochemicals. Importantly, it is still necessary to further investigate the effects of these nanodispersions on the soil microbiota and on the growth of plants.

### Supplementary Information

**Additional file 1: Figure S1.** *T. harzianum *10BR1, *F. oxysporum*, and *F. verticillioides*, from left to right, after 48 hours of incubation at a final concentration of 0.110 g.mL^-1^ of nanodispersions. (1) Treatment with water, (EXT) treatment with extract (0.010 g.mL^-1^) in water, (3) treatment with PLE, and (4) treatment with PZLE.

## Data Availability

Data will be made available on request.
