# Supplementary material for: Effects of Larrea nitida nanodispersions on the growth inhibition of phytopathogens
Source: AMB Express. 2023 Sep 21;13:98. doi: 10.1186/s13568-023-01605-z (PMC10514021; doi:10.1186/s13568-023-01605-z)
Supplement: Supplementary file 1 — Additional file 1: Figure S1. T. harzianum 10BR1, F. oxysporum, and F. verticillioides, from left to right, after 48 hours of incubation at a final concentration of 0.110 g.mL-1 of nanodispersions. (1) Treatment with water, (EXT) treatment with extract (0.010 g.mL-1) in water, (3) treatment with PLE, and (4) treatment with PZLE. [file 13568_2023_1605_MOESM1_ESM.docx]

**Supporting Information**

**Effects of *Larrea nitida* nanodispersions on the growth inhibition of phytopathogens**

Felipe Rochaª, Rodrigo José Nunes Calumby^a^, Laura Svetaz^b^, Maximiliano Sortino^b^, Márcia Cristina Teixeira Ribeiro Vidigal^c^, Valeria Alina Campos Bermudez^a*^, Sebastián Pablo Rius^a*^

ªCentro de Estudios Fotosintéticos y Bioquímicos (CEFOBI-CONICET), Universidad Nacional de Rosario, Suipacha 531, S2002LRK, Argentina

^b^Farmacognosia, Facultad de Ciencias Bioquímicas y Farmacéuticas, Universidad Nacional del Rosario, Suipacha 531, CP 2000, Rosario, Argentina

^c^Food Science Department, Federal University of Vicosa, Peter Henry Rolfs Avenue, 36570-900 Vicosa, MG, Brazil

*Corresponding authors: [campos@cefobi-conicet.gov.ar](mailto:campos@cefobi-conicet.gov.ar) (V. A. C. Bermudez), [rius@cefobi-conicet.gov.ar](mailto:rius@cefobi-conicet.gov.ar) (S. P. Rius).


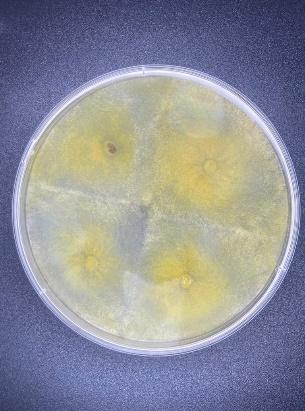

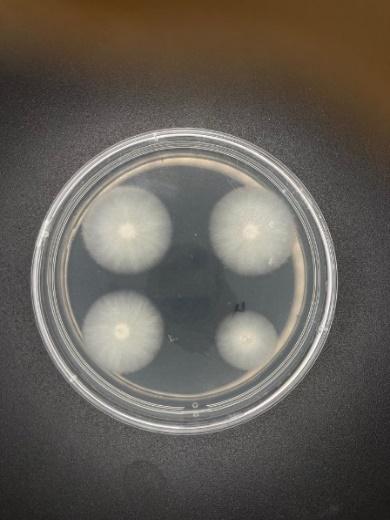

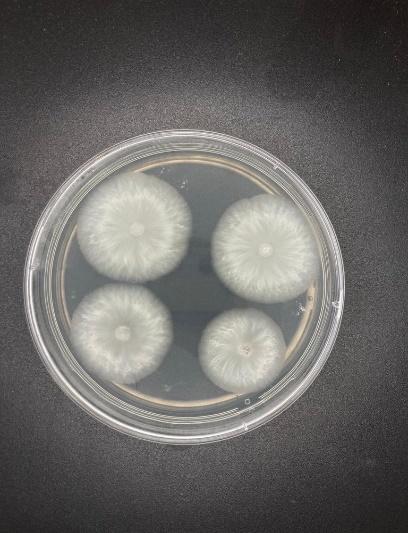


EXT

1

4

3

Fig. S1 –*T. harzianum* 10BR1, *F. oxysporum*, and *F. verticillioides*, from left to right, after 48 hours of incubation at a final concentration of 0.110 g.mL^-1^ of nanodispersions. (1) Treatment with water, (EXT) treatment with extract (0.010 g.mL^-1^) in water, (3) treatment with PLE, and (4) treatment with PZLE
